# Elevated herpesvirus antibody levels linked to schizophrenia and bipolar disorder

**DOI:** 10.1192/j.eurpsy.2024.148

**Published:** 2024-08-27

**Authors:** D. Andreou, N. E. Steen, K. N. Jørgensen, T. Ueland, L. A. Wortinger, L. Mørch-Johnsen, R. H. Yolken, O. A. Andreassen, I. Agartz

**Affiliations:** ^1^Department of Psychiatric Research, Diakonhjemmet Hospital; ^2^NORMENT, Division of Mental Health and Addiction, Oslo University Hospital & Institute of Clinical Medicine, University of Oslo, Oslo, Norway; ^3^Centre for Psychiatry Research, Department of Clinical Neuroscience, Karolinska Institutet & Stockholm Health Care Services, Stockholm Region, Stockholm, Sweden; ^4^Research Institute of Internal Medicine, Oslo University Hospital, Rikshospitalet, Oslo, Norway; ^5^Stanley Division of Developmental Neurovirology, Department of Pediatrics, Johns Hopkins University School of Medicine, Baltimore, MD, United States

## Abstract

**Introduction:**

Previous research has implicated herpes simplex virus 1 (HSV1) and cytomegalovirus (CMV) in severe mental illness (SMI) with conflicting results. Both pathogens have high universal seroprevalence, are neurotropic and after the primary infection typically establish a persistent latent infection with periodic reactivations. Increased immunoglobin G (IgG) concentrations are considered to be attributable to an increased infection severity with more frequent reactivations or host immune system alterations.

**Objectives:**

We assessed the HSV1 and CMV IgG concentrations in previously infected (seropositive) patients with SMI and healthy controls (HC). We hypothesized that seropositive patients would show higher IgG concentrations than seropositive HC.

**Methods:**

We included 765 patients, 515 with schizophrenia (SZ) and 250 with bipolar disorder (BP), and 541 HC. HSV1 and CMV IgG seropositivity and concentrations were measured with immunoassays. 355 patients, mean age 33 years, 45% females, and 238 HC, mean age 35 years, 44% females, were HSV1 seropositive (HSV1+) while 447 patients, mean age 33 years, 50% females, and 296 HC, mean age 34 years, 47% females, were CMV seropositive (CMV+). In our main analysis among seropositive participants, we investigated the main effect of patient/control status on HSV1 and CMV IgG concentrations.

**Results:**

There were no significant differences in CMV or HSV1 seropositivity frequencies between patients with SZ, patients with BP and HC. Among seropositive participants, patients had higher HSV1 (p<0.001) and CMV (p=0.018) IgG concentrations than HC; stratifying by diagnosis, both patients with SZ (p=0.001) and patients with BP (p=0.001) had higher HSV1 IgG concentrations than HC, while patients with SZ, but not BP, had higher CMV (p=0.045) IgG concentrations than HC (Image). For HSV1, higher IgG concentrations were associated with higher general (p=0.017), negative (p=0.041) and positive (p=0.028) psychotic symptom scores.

**Image:**

**Figure:**
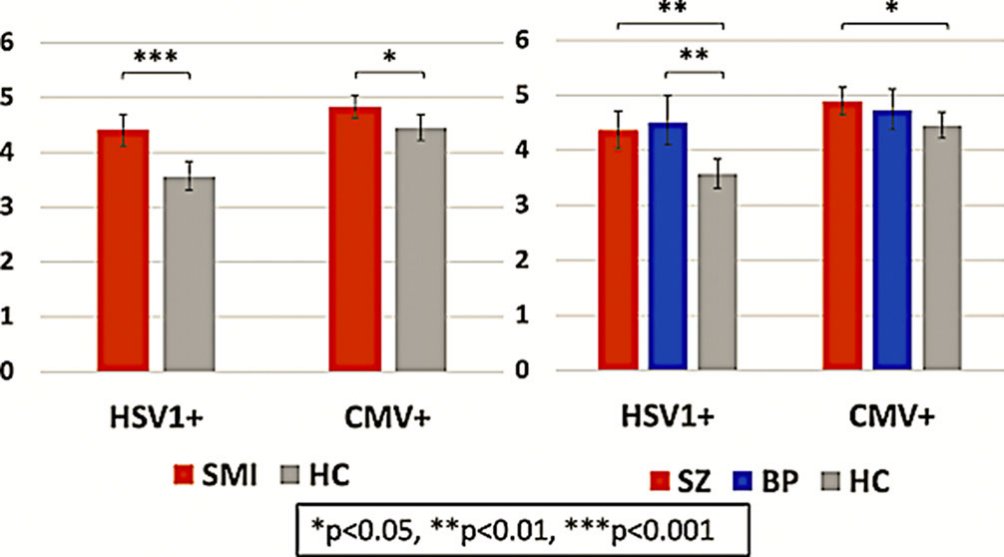

**Conclusions:**

Seropositive patients with SMI showed higher HSV1 and CMV IgG concentrations than seropositive HC suggesting that patients suffer a more severe infection or exhibit an altered immune response when contracting the pathogens. For HSV1, higher IgG concentrations were linked to more psychotic symptoms.

**Disclosure of Interest:**

D. Andreou: None Declared, N. E. Steen: None Declared, K. N. Jørgensen: None Declared, T. Ueland: None Declared, L. Wortinger: None Declared, L. Mørch-Johnsen: None Declared, R. Yolken: None Declared, O. Andreassen Consultant of: Consultant to HealthLytix, Speakers bureau of: Received speaker’s honorarium from Lundbeck and Sunovion, I. Agartz Speakers bureau of: Received speaker’s honorarium from Lundbeck

